# Prevalence, Reasons, Predictors, Perceived Effects, and Regulation of Alcohol Use among Children in Ghana

**DOI:** 10.1155/2023/9032348

**Published:** 2023-07-18

**Authors:** Sylvester Kyei-Gyamfi, Nii Wellington, Frank Kyei-Arthur

**Affiliations:** ^1^Department of Children, Ministry of Gender, Children and Social Protection, Accra, Ghana; ^2^Development Education Centre–Ghana, Box M103, Ministries, Accra, Ghana; ^3^Department of Environment and Public Health, University of Environment and Sustainable Development, Somanya, Eastern Region, Ghana

## Abstract

Early initiation of alcohol consumption increases the risk of alcohol dependence and adverse health outcomes. Consequently, nations have enacted laws to make alcohol unlawful to be purchased by, sold to, or used in public by children. This study examined the lifetime prevalence of alcohol use among children and their reasons for consuming alcohol. In addition, the study investigated predictors of alcohol consumption and the effects of alcohol use on children. Finally, it examined the effectiveness of measures in place for regulating the sale and use of alcohol by children in the country. A total of 5024 children between the ages of 8 and 17 were sampled across the ten regions of Ghana using a cross-sectional convergent parallel mixed method. Children were interviewed with a semi-structured questionnaire, while focus group discussions were held with children, parents, and key informants. Lifetime prevalence of alcohol consumption was measured by “have you ever taken alcohol?”. The study revealed that lifetime alcohol consumption was less prevalent (6.6%) among children. Sex, age, and region of residence were significant predictors of lifetime alcohol use among children. More than half of the children who reported ever taking alcohol were first introduced to drinking by friends, and more than six in ten children claimed having been intoxicated after drinking alcohol. The findings further revealed that efforts to control the sale and consumption of alcohol by children have proven difficult despite the existence of laws, policies, and national regulatory structures. While regulations on alcohol sales and consumption have been difficult to implement in rural areas, they have been successful in urban areas because institutions there ensure rigorous adherence to the regulations. The study encourages national organizations with responsibility for child protection and development to step up their regulation, investigation, and information-sharing efforts to discourage and limit children from purchasing and consuming alcohol.

## 1. Introduction

When a person drinks alcohol while under the legal drinking age, it is referred to as underage drinking. Early alcohol usage is linked to future dependency, health problems, and poor ability to interact with others [1]. Globally, about three in ten (26.5%) children between the ages of 15 and 19 have been estimated to have ever consumed alcohol [2].

In several nations, alcohol use starts before the age of 15; hence, some nations have passed laws making it illegal for children (persons under the age of 18) to buy, sell, or consume alcohol in public [2, 3]. Ghana is among the nations with legislation prohibiting the consumption of alcohol by children. The Liquor Licensing Act of 1970 (Act 331) establishes an age limit of 18 or older for alcohol consumption in Ghana [4]. To regulate alcohol usage and fight underage drinking nationwide, Ghana's government also established the National Alcohol Policy and the Narcotics Control Board (NACOB).

Oshodin [5] highlighted that males were more likely than females to drink alcohol in traditional African societies. According to the author, indulgence in excessive consumption of alcohol and intoxication received severe punishment, and women and children were discouraged from taking alcohol. However, alcohol drinking habits are changing in many African societies as a result of societal changes, economic progress, and the expansion of the alcohol industry [6]. The number of children who engage in alcohol consumption has increased as a result of these changes, and Ghana is no exception.

Studies in Ghana show a rise in the prevalence and involvement of children in alcohol usage [7, 8]. The 2016 Multiple Indicator Cluster Survey (MICS), for instance, indicates that 22% of males and 19% of females between the ages of 15 and 19 had, at some point, drunk alcohol [9]. Children abusing alcohol is a public health issue that has repercussions for the country's young population's welfare and development [10]. Bonnie [11] revealed that underage drinking has a number of negative effects, such as accidental death and injury, poor academic performance, and strained relationships as a consequence of alcoholism and addiction. Early alcohol consumption is also associated with an increased likelihood of adult alcohol dependence and alcohol use disorders [12, 13]. In addition, drinking alcohol has been linked to an increase in youth-related violence [2].

Although drinking alcohol has detrimental effects on children, most studies conducted in Ghana have only looked at the general population [14–17]. In addition, the few studies on children and young people are not representative of the entire country [7, 8, 18]. For instance, a study by Hormenu et al. [7] among in-school children aged 10–15 in the central region found that 42.3% had ever consumed alcohol. Also, a study by Amoah et al. [18] among senior secondary students in Bono East region in Ghana found that 22.2% of students currently consume alcohol, while 27.9% had ever consumed alcohol. Thus, there are limited studies focusing on alcohol consumption among children using nationally representative data. Studying alcohol consumption among children using nationally representative data will help researchers and policymakers to understand better the phenomenon of alcohol consumption among children, which will help to develop effective interventions to address the phenomenon.

In light of this, the study examined the lifetime prevalence of alcohol consumption among children and the reasons underlying their alcohol use. The study additionally investigated predictors of alcohol usage and the effect of alcohol use on children. The effectiveness of policies in place for regulating alcohol sale and use by children in the country was also examined.

### 1.1. Hypothesis Development

Previous studies have shown that the prevalence of alcohol consumption among children differs by socio-demographic characteristics such as sex and age [2, 7, 19, 20]. For instance, previous studies have reported that alcohol consumption is more prevalent among male children than female children [8, 9, 21]. In addition, research has found a positive relationship between children's age and alcohol intake [9, 22, 23]. Based on these studies, this study tested two hypotheses as listed as follows:

  Hypothesis 1 (H1): males are more likely to have ever consumed alcohol than females
  Hypothesis 2 (H2): older children are more likely to have ever consumed alcohol than younger children

## 2. Materials and Methods

### 2.1. Study Design and Sampling Procedure

This study used a cross-sectional convergent parallel mixed-method design to understand the phenomenon of alcohol consumption among children aged 8–17. For the purpose of this study, persons aged 8–17 are defined as children. A convergent parallel mixed-method design simultaneously gathers quantitative and qualitative data to understand a social phenomenon [24]. The study was conducted by the Department of Children (DOC) of the Ministry of Gender, Children and Social Protection (MoGCSP) in the 10 regions of Ghana. A multistage sampling procedure was used to sample children aged 8–17. In the first stage, 20% of districts in each of the country's 10 regions were chosen based on child protection issues such as child marriage and child labor. In 2018, Ghana had 216 districts; 43 districts (20%) were selected. In the second stage, 15 enumeration areas (EAs) were randomly selected in each of the 43 districts. In total, 645 EAs were selected. In the third stage, children aged 8–17 in each EA were selected proportionate to the district's size. It is worthy of note that in each household, only one child aged 8–17 was selected.

#### 2.1.1. Quantitative Data

For the quantitative data, a semi-structured questionnaire was administered face-to-face to children aged 8–17. In total, 5,024 children aged 8–17 were interviewed.

#### 2.1.2. Qualitative Data

Ten focus group discussions (FGDs) with children between the ages of 8 and 17 were conducted as part of the qualitative data collection. The FGDs were held in ten towns, each in a region, in convenient sites decided upon by the participants. Each FGD had a maximum of 14 participants. Also, ten FGDs were held with parents of children aged 8–17 at convenient places in ten different towns. In addition, fifty key informant interviews (KIIs) were conducted at convenient locations with representatives from a range of Government Ministries, Departments, and Agencies (MDAs), and Metropolitan Municipal and District Assemblies (MMDAs).

### 2.2. Study Setting

In order to fulfill Ghana's international reporting requirements under the Convention on the Rights of the Child (CRC), the Government of Ghana through MoGCSP conducted a study on the living circumstances of children in Ghana. Ghana is a nation in sub-Saharan Africa, bordered to the north by Burkina Faso, to the south by the Gulf of Guinea, to the east by Togo, and to the west by Cote d'Ivoire. Ghana's population was estimated at 30.8 million as of 2021 by the Ghana Statistical Service (GSS) [25], with 6.8 million (22.1%) of the population being children under the age of 17. Ghana now has 16 administrative regions, an increase of 6 regions from 2018, the year the study's data were gathered.

### 2.3. Measurement and Variables

The dependent variable for the study was the lifetime prevalence of alcohol consumption, which was measured with the question, “Have you ever taken alcohol?”, with the response options being 1 = yes, and 2 = no.

The independent variables for this study included sex (male and female), age (8–10 years, 11–13 years, and 14–17 years), educational attainment (not in school, primary, junior high school (JHS), and senior high school (SHS)/vocational/technical/commercial, and tertiary), religion (Christian, Muslim, Traditional, and no religion), and region (Greater Accra, Central, Western, Eastern, Volta, Ashanti, Brong Ahafo, Northern, Upper East, and Upper West).

Alcohol consumption was also measured qualitatively by asking children the types of alcohol children consumed and where they purchased alcoholic beverages. In addition, key informants were asked about measures by the Government of Ghana to protect children from substance abuse, especially regulations on alcohol sales and consumption by children. Parents were also asked about the challenges associated with the regulations on alcohol sales and consumption.

### 2.4. Data Collection

This study is a part of a comprehensive study by the MoGCSP on the living circumstances of children in Ghana [26]. Living and household arrangements, education and training, social amenities, children's rights, health and healthcare, sexual and reproductive health, usage of the internet and social media, child employment, and children's goals and aspirations were some of the subjects covered by the comprehensive study.

It took seven months to gather the data, starting in April 2018. A one-day workshop was held to train field assistants on how to interview key informants and conduct FGDs with children aged 8–17 as well as administer the semi-structured questionnaire to children aged 8–17.

### 2.5. Statistical Analysis

Descriptive analyses (univariate analyses), such as frequencies and percentages, were used to describe the children socio-demographic characteristics, lifetime alcohol consumption, ever been drunk, initiation into alcohol consumption, frequency of alcohol use, reasons for drinking alcohol, and perceived effects of alcohol consumption. Pearson's Chi-square and Fisher's exact tests (bivariate analyses) were employed to examine the association between the sociodemographic characteristics of respondents and the dependent variable (ever taken alcohol). Fisher's exact test is recommended when a contingency table cell has an expected frequency of less than 5 [27–29]. In addition, Pearson's Chi-square and Fisher's exact tests were employed to examine the association between reasons for drinking alcohol and the sex of children and the perceived effects of alcohol consumption and the sex of children, respectively.

A binary logistic regression (multivariate analysis) was performed to identify the predictors of children's lifetime alcohol usage. All the quantitative data analyses (univariate, bivariate, and multivariate analyses) were carried out using the Statistical Package for the Social Sciences (SPSS) version 26. All variables were statistically significant at 95% confidence interval (*P* < 0.05).

All interviews were audio-taped and transcribed verbatim from the local languages (such as Twi, Ewe, and Dagbani) to English. Thematic analysis was used to analyze all transcripts. All transcripts were read several times to get a general sense of children's experiences. Statements made by respondents that were pertinent to the study's objectives were given codes. Similar codes were grouped into sub-themes, and similar sub-themes were grouped into themes.

### 2.6. Ethical Considerations

The National Child Protection Committee (NCPC) of the DOC of MoGCSP gave approval for the study. The data collected for this study are part of a national monitoring exercise to evaluate the effectiveness child rights adherence in Ghana. The data collection process adhered to the Helsinki Declaration's principles. Children's rights were observed by protecting their privacy and anonymizing the collected data. All respondents (children, parents, officials of parents, and officials of MDAs and MMDAs) who participated in the study gave informed consent before being interviewed.

## 3. Results

### 3.1. Socio-Demographic Characteristics of Respondents

Table 1 shows the socio-demographic characteristics of the children sampled. Males made up more than half of the participants (51.1%). Most children (75.1%) were Christians, and a higher proportion were aged between 14 and 17 (43.7%). More children aged between 14 and 17 had significantly (*P* ≤ 0.001) ever consumed alcohol than those aged between 8 and 10 and those aged 11 and 13. More children were in primary school (42.1%). However, a higher proportion of children in SHS had significantly (*P* ≤ 0.001) ever consumed alcohol than those in other educational categories. Also, more children resided in the Brong Ahafo region (14.7%). However, more children who resided in the Volta region had significantly (*P* ≤ 0.001) ever consumed alcohol than those in other regions.

### 3.2. Ever Taken Alcohol and Ever Been Drunk after Consuming Alcohol

Table 2 displays the responses from study participants about alcohol usage. According to Table 2, nearly seven percent (6.6%) of children have ever consumed alcohol. Among those who had ever taken alcoholic beverages, the majority (63.4%) got drunk after consumption.

### 3.3. Predictors of Alcohol Consumption among Children

Table 3 shows the variables that predict lifetime alcohol consumption among children. Respondents not in school and those in primary schools were merged for educational attainment since they did not contain enough cases for the multivariate analysis. Table 3 revealed that male children (AOR = 1.272; Wald test = 4.200; 95% CI = 1.011–1.601; *P*=0.040), children aged 11–13 (AOR = 2.666; Wald test = 11.000; 95% CI = 1.493–4.758; *P*=0.001), and children aged 14–17 (AOR = 8.088; Wald test = 33.160; 95% CI = 3.971–16.476; *P* ≤ 0.001) were significantly more likely to have ever consumed alcohol.

However, children residing in the Eastern (AOR = 0.320; Wald test = 13.592; 95% CI = 0.175–0.587; *P* ≤ 0.001) and Northern regions (AOR = 0.565; Wald test = 4.236; 95% CI = 0.329–0.973; *P*=0.040) were significantly less likely to have ever consumed alcohol.

### 3.4. Initiation into Alcohol Consumption, Reasons, and Perceived Effects of Alcohol on Children

Children were asked when they first initiated alcohol consumption (Table 4). Table 4 shows that more than half (53.5%) of children were initiated into alcohol consumption by friends. In contrast, 17.4% were initiated by elders in their family or household.

According to the respondents (Table 4), children consume alcohol for varied reasons, including group culture (32.1%), boost confidence (21.6%), whet appetite for meals (18.6%), and taking as medicine (15.0%). There is a significant association between the initiation of alcohol consumption and the sex of children (*P*=0.037). A higher proportion of male children reported group culture (51.4%), boost confidence (51.4%), and whet appetite for meals (67.7%) as their main reasons for consuming alcohol than the females. However, more females mentioned taking alcohol as medicine (58.0%) as their main reason for consuming alcohol than males (42.0%).

When asked about the effects of drinking alcohol, 39.3% believed it causes children to perform poorly in school. In contrast, 36.0% believed it causes people to become deviants or misconduct themselves. In addition, respondents reported that alcohol consumption results in accidents and fatalities (9.3%), makes a person confident/stronger (7.2%), helps individuals forget their troubles (7.2%), and increases the likelihood of illness infection (0.9%).

### 3.5. Regulation of Alcohol Sale and Use among Children

Interviews with the officials of the Narcotic Control Commission (NACOC) revealed that measures in place for protecting children from substance abuse include the enactment of the Narcotic Control Commission Act, 2020 (Act 1019), which is an amendment of the Narcotics Drugs (Control, Enforcement and Sanctions) Law 1990 (PNDCL 236). The law bans narcotic drugs and also led to the establishment of the Commission, which has been successful in curbing the flow of drugs into the country. NACOC officials also mentioned that Ghana has signed and ratified various United Nations Conventions and Protocols on drugs. These include the 1961 Single Convention, 1971 Convention on Psychotropic Substances, 1972 Protocol Amending the 1961 Single Convention, and 1988 Convention against Illicit Trafficking of Narcotic Drugs and Psychotropic Substances. They went on to indicate that Ghana endorsed the international treaties to deal with issues related to drug control and other associated substances affecting young people, particularly children. Periodically, the NACOC and other state organizations, such as the Department of Social Welfare and the National Commission for Civic Education (NCCE), conduct public awareness programs in educational facilities, and communities to raise awareness of the negative impacts of drug and alcohol abuse. The followings are quotes from the NACOC officials to buttress the point:

Campaigns to raise community awareness of the negative consequences of alcohol abuse are frequently carried out by the National Commission for Civic Education (NCCE), NACOB and the Department of Children and the Department of Social Welfare under the Ministry of Gender and Children and Social Protection. (KII 5).

However, key informants explained that NACOB and other state institutions experience several challenges, including inadequate funds, affecting its capacity to fight alcohol and drug-related issues in the country. The followings are the views expressed by key informants:

The government finances NACOB and sometimes gets partnership funding from donors. However, we have inadequate funding, a major concern that needs to be addressed to enable us to carry out our respective mandates for effective outcomes in our operations. (KII 1).

Key informants at the Accra Psychiatric Hospital also highlighted that no therapy facility in the country is solely dedicated to providing service for children with alcohol and drug-related issues.

Most of the annual cases at the Accra Psychiatric Hospital are due to drug misuse among young people [including children]. The fact that most therapy facilities in the country only accept adults is the main cause for concern. It is really concerning that there is now no facility in the whole country for children. (KII 3).

Regarding alcohol sale and consumption regulation, key informants narrated that the Ministry of Health has adopted a National Alcohol Policy to harmonize all laws that exist to control the production, distribution, sale, promotion, and consumption of alcohol in the country. By regulating the production, distribution, sale, promotion, and consumption of alcohol, the new policy seeks to reduce the negative consequences that alcohol abuse has on individuals and their families. A key informant had this to say:

The new Policy [National Alcohol Policy], which the Ministry of Health (MOH) and the WHO unveiled, will encourage and promote abstinence [from alcohol consumption], lessen hazardous alcohol consumption, adhere to best practices around the world, and urge the government to take the lead in guaranteeing full compliance. (KII 2).

The FGDs with parents revealed that regulating the sale and consumption of alcohol by children in rural areas of the country is problematic. Parents in the Western and Eastern regions claimed that because the law prohibiting the sale of alcohol to minors is not strictly enforced, rural areas have a higher prevalence of underage drinking than urban areas. The use of alcohol by young people, especially children, has also been linked to disrespect, criminal activity, and other aberrant behaviors, according to parents. One parent explained:

Many of the children around here are alcoholics. They drink and misbehave in their homes and on the streets, and when adults dare to object, they will be beaten up or insulted. Due to their alcoholism, many parents no longer have complete control over their children in the community. (FGD 7).

FGDs with children confirmed that children consumed local alcoholic beverages, including palm wine and “Akpeteshie” (a popular locally produced alcoholic beverage in Ghana). Children often purchase alcoholic beverages from neighborhood bars, especially in rural areas, and bars in rural areas have no limitations on selling alcoholic beverages to children compared to urban areas. One key informant expressed:

All the drinking bars allow children to enter freely and purchase alcohol. Who they sell to is irrelevant to the bar owners. Their sole focus is on increasing sales. They refuse to heed the summons, despite the complaints of their parents and elders. (FGD 20).

## 4. Discussion

This study aimed to examine children lifetime prevalence of alcohol consumption and their reasons for alcohol consumption. The study also investigated predictors of children lifetime alcohol use and the effects of alcohol consumption on children. It furthermore examined the effectiveness of policies in place for regulating alcohol sale and use among children in Ghana.

The study found that children lifetime prevalence of alcohol consumption was 6.6%. Children lifetime prevalence of alcohol consumption in this study is lower than prevalence found previous studies from Ghana [7–9, 18], Nigeria [19, 21], and Iran [30]. For instance, Roshanfekr et al.'s [30] study among street children in Iran found that 16.6% had ever consumed alcohol. In Ghana, a study by Hormenu et al. [7] among in-school adolescents found that 42.3% of adolescents had ever consumed alcohol. The discrepancies in the prevalence of alcohol consumption could be attributed to varied factors, including differences in the sample population, measurement of alcohol consumption, and the studies not covering the whole country. For instance, previous studies on alcohol consumption among children in Ghana focused on selected regions in Ghana, namely, Central [7], Eastern [8], and Bono East [18] regions, than the entire country. Also, Alex-Hart et al.'s [19] study was among secondary school students, while Roshanfekr et al.'s [30] study was among street children. In terms of measurement of alcohol consumption, Hormenu et al.'s [7] study in Ghana measured alcohol consumption among in-school adolescents using how old respondents were when they had their first alcoholic drink.

The study also found that males are more likely to have ever consumed alcohol than females. This finding supports the first hypothesis that males are more likely to have ever consumed alcohol than females. The finding is consistent with prior studies [9, 19, 21], showing that alcohol consumption is more prevalent among males than females. Cultural expectations of masculinity may explain the high prevalence of alcohol consumption among males [5, 31, 32]. Culturally, alcohol consumption is acceptable for men but disapproved of for women [5]. However, this finding contrasts with previous studies in Ghana [8] and the United States [20], which found a high prevalence of alcohol consumption among females than males.

Regarding the age of children, our study found that there is a positive association between alcohol consumption and children's age. Thus, children aged 14–17 years are more susceptible to alcohol consumption than children aged 8–10 years. This finding confirms the second hypothesis that older children are more likely to have ever consumed alcohol than younger children. This finding corroborates earlier studies which found that alcohol consumption increases with age [2, 9, 22]. For example, a study by Aboagye et al. [23] among Ghana's tertiary students found a positive relationship between alcohol consumption and the age of students. Similarly, a study by Hormenu et al. [7] among in-school adolescents found that older adolescents (14-15 years) consumed more alcohol than younger adolescents (12-13 years). A plausible explanation is that as children grow, they are increasingly exposed to advertisements on alcoholic beverages, which may entice them to consume them.

The results also indicated that 1.4% of children aged between 8 and 10 had ever consumed alcohol. Zucker et al.'s [33] study found that early alcohol usage (before the age of 12) is linked with an increased risk of developing alcohol-related challenges during adulthood. Dunn and Goldman's study [34] highlighted that children's negative perceptions of alcohol and its effects change as they age. Older children, by extrapolation, have a higher alcohol consumption urge than younger children. This points to the importance of early interventions to discourage the early initiation of alcohol to forestall the development of alcohol-related challenges in later life.

Regarding the region of children's residence, the findings indicate that children from the Eastern and Northern regions are significantly less likely to have ever consumed alcohol than children from the Greater Accra region. A plausible explanation is that the Greater Accra region, which has Accra, the capital of Ghana, is often the epicentre of programs and advertisements on alcoholic beverages. The exposure of children in Greater Accra to programs and advertisements on alcoholic beverages may explain why they are more likely to have ever consumed alcohol. Studies have linked increased advertisements on alcoholic beverages with increased consumption of alcohol [35–37].

Our results also highlighted that more than half of children (53.5%) were influenced by friends to initiate alcohol consumption. This result corroborates other studies which found peer influence as a major predictor of alcohol consumption among children [38–40]. Proximately 17% of children were influenced by their older family members to initiate alcohol consumption, which supports the findings of earlier studies [5, 31]. Our result also showed that 17% of children consumed their first alcohol at social gatherings, corroborating previous research findings [41]. Twelve percent of children had their first alcoholic beverages at drinking bars, and this phenomenon is more prevalent in rural areas [42, 43]. This result is not unexpected given the wide variety of drinking bars/pubs in Ghana [44], and it is common for adults and parents to send children to purchase alcoholic beverages at these drinking places [45].

In addition, this study found that alcohol consumption was part of children's group culture, corroborating previous studies which found peer group influence as a significant determinant of children attitudes and actions [38]. When some members of a group consume alcohol, it is possible other members may be influenced to initiate alcohol consumption due to group culture.

Some children were motivated to consume alcoholic beverages to boost their confidence (21.6%), whet their appetite for meals (18.6%), and as a medication for the treatment of their ailments (15.0%). This finding is unsurprising since advertisements on alcoholic beverages highlight these attributes, influencing children to consume alcohol [46]. Studies have also documented the use of alcohol in preparing herbal medications to treat ailments [47, 48].

Furthermore, 7.5% of children reported that drinking alcohol was a ritual in their groups or homes, supporting other studies [41]. Also, 5.1% of children reported consuming alcohol to forget their worries. This finding supports previous studies, which found that adults sometimes consume alcohol to forget about problems/issues [49–51]. This finding highlights the need for health practitioners and policymakers to educate children on active coping strategies for their daily challenges.

Respondents mentioned varied effects of alcohol consumption, including negative impacts on academic achievement, involvement in delinquent behavior, occurrences of accidents and fatalities, enhanced self-assurance, forgetting challenges, and heightened susceptibility to contracting diseases. Despite children being knowledgeable about the negative effects of alcohol consumption, there is a need to strengthen public education on alcohol consumption to reduce the prevalence of children's alcohol consumption.

Our results highlighted the availability of regulations, policies, and state agencies to regulate children's alcohol consumption and its effects. Despite these measures (legislations, policies, and state agencies), the sale of alcoholic beverages to children and children alcohol consumption is a common phenomenon. Also, there is a weak enforcement of regulations on selling alcoholic beverages to children in rural areas, making those children more susceptible to consuming alcohol. This finding is similar to a study by Senya and Floyd [52] in Ghana, which found that most sellers of alcoholic beverages (85%) were willing to sell alcohol to children to increase their sales, although they were aware it was unlawful.

Furthermore, alcohol intoxication made children in rural areas to exhibit negative behavioral outcomes, including disrespect, violence, rebellion, and disregard for the law. This result supports other studies which reported alcohol-related problems, including rebellion [53], violence [54], unruliness, and disrespect for authority [55, 56]. Inferring from the results, children who reside in rural areas are a vulnerable population to indulge in underage alcohol consumption. Hence, policymakers and researchers need to focus on this population to better understand their needs to enhance their health and general wellbeing due to the adverse effects of underage alcohol consumption on children. In addition, policymakers should ensure the enforcement of regulations on alcohol sales and use among children, especially in rural areas. In addition, organizations mandated to deal with the menace of underage alcohol consumption in the country should strengthen their efforts in public education regarding the adverse consequences of alcohol consumption on children and the general population.

## 5. Limitations

This study has some limitations. Children were inquired regarding their history of alcohol consumption. Due to the perception of underage alcohol consumption as deviant and illegal for individuals below the age of 18, there is a likelihood that children may provide inaccurate or incomplete accounts of their involvement in such activities. Also, the study was cross-sectional. Hence, causality cannot be established. Third, studies have identified vital variables that predict alcohol consumption, including peer and parental use of alcohol, household wealth quintile, and academic adjustment problems [16–18, 23]. However, these variables were not included in the binary logistic regression since they were unavailable in the dataset. Despite these limitations, the findings of this nationally represented study deepen health practitioners' and policymakers' understanding of children's alcohol consumption in Ghana, which will aid in designing appropriate interventions to curb the phenomenon.

## 6. Conclusions

Our results showed that children lifetime alcohol consumption was less than one-tenth (6.6%). Lifetime alcohol consumption was prevalent among older children (14–17 years), children with senior high school education, and males. Again, friends were a major determinant of alcohol consumption among children. Most children got drunk after consuming alcohol, and boosting confidence was the main facilitator of alcohol consumption among children. The findings of the study demonstrated that the government's efforts to stop the sale and consumption of alcohol by children have been ineffective.

Furthermore, the sex of children, age of children, and region of residence were significant predictors of alcohol consumption among children aged between 8 and 17. Policymakers should target these characteristics when developing interventions to reduce alcohol consumption among children.

## Figures and Tables

**Table 1:** Socio‐demographic characteristics of respondents.

Variables	*n* (%)	Ever taken alcohol		
		Yes *n* (%)	No *n* (%)	*P* value
*Sex*				
Male	2566 (51.1)	181 (7.1)	2385 (92.9)	0.215
Female	2458 (48.9)	152 (6.2)	2306 (93.8)	

*Age*				
8–10	1330 (26.5)	19 (1.4)	1311 (98.6)	≤0.001
11–13	1497 (29.8)	62 (4.1)	1435 (95.9)	
14–17	2197 (43.7)	252 (11.5)	1945 (88.5)	
Mean ± Standard deviation	12.9 ± 3.0			

*Educational attainment*				
Not in school	27 (0.5)	0 (0.0)	27 (100.0)	≤0.001^*∗*^
Primary	2116 (42.1)	50 (2.4)	2066 (97.6)	
JHS	1584 (31.5)	143 (9.0)	1441 (91.0)	
SHS	1183(23.5)	130 (11.0)	1053 (89.0)	
Tertiary	114 (2.3)	10 (8.8)	104 (91.2)	

*Religion*				
Christian	3771 (75.1)	260 (6.9)	3511 (93.1)	0.424
Muslim	1083 (21.6)	60 (5.5)	1023 (94.5)	
Traditional	103 (2.1)	8 (7.8)	95 (92.2)	
No religion	67 (1.3)	5 (7.5)	62 (92.5)	

*Region*				
Greater Accra	360 (7.2)	31 (8.6)	329 (91.4)	≤0.001
Central	474 (9.4)	39 (8.2)	435 (91.8)	
Western	377 (7.5)	25 (6.6)	352 (93.4)	
Eastern	598 (11.9)	18 (3.0)	580 (97.0)	
Volta	595 (11.8)	56 (9.4)	539 (90.6)	
Ashanti	707 (14.1)	48 (6.8)	659 (93.2)	
Brong Ahafo	738 (14.7)	43 (5.8)	695 (94.2)	
Northern	722 (14.4)	35 (4.8)	687 (95.2)	
Upper East	237 (4.7)	22 (9.3)	215 (90.7)	
Upper West	216 (4.3)	16 (7.4)	200 (92.6)	

^*∗*^Fisher exact test.

**Table 2:** Distribution of ever taken alcohol and ever been drunk after consuming alcohol.

Variables	*n*	%
*Ever taken alcohol*		
Yes	333	6.6
No	4691	93.4

*Ever been drunk after consuming alcohol*		
Yes	211	63.4
No	122	36.6

**Table 3:** Predictors of alcohol consumption among children.

Variables	AOR	Wald	95% CI	*P* value
*Sex*				
Male	1.272	4.200	1.011–1.601	0.040
Female (RC)				

*Age*				
8–10 Years (RC)				
11–13 Years	2.666	11.000	1.493–4.758	0.001
14–17 Years	8.088	33.160	3.971-16.476	≤0.001

*Education*				
Not in school/primary	1.307	0.376	0.555–3.075	0.540
JHS	1.542	1.532	0.777–3.062	0.216
SHS/vocational/technical/commercial	1.411	0.978	0.713–2.792	0.323
Tertiary (RC)				

*Religion*				
Christian	0.983	0.001	0.383–2.525	0.971
Muslim	0.951	0.010	0.355–2.548	0.921
Traditional	0.984	0.001	0.298–3.249	0.979
No religion (RC)				

*Region*				
Greater Accra (RC)				
Central	0.818	0.607	0.493–1.357	0.436
Western	0.630	2.621	0.360–1.102	0.105
Eastern	0.320	13.592	0.175–0.587	≤0.001
Volta	1.052	0.044	0.655–1.689	0.834
Ashanti	0.739	1.523	0.457–1.195	0.217
Brong Ahafo	0.738	1.460	0.451–1.208	0.227
Northern	0.565	4.236	0.329–0.973	0.040
Upper East	1.044	0.021	0.579–1.883	0.885
Upper West	0.860	0.202	0.447–1.658	0.653

AOR = adjusted odds ratio; RC = reference category; CI = confidence interval.

**Table 4:** Initiation into alcohol consumption, reasons for drinking alcohol, and perceived effects of alcohol on children by sex of children.

Response	Frequency (%)	Sex of children		
		Male	Female	*P*-value
		Frequency (%)	Frequency (%)	
*Initiation into alcohol consumption*				
Friends	178 (53.5)	96 (53.9)	82 (46.1)	0.971
Elders in family/household	58 (17.4)	33 (56.9)	25 (43.1)	
Party/ceremony	57 (17.1)	30 (52.6)	27 (47.5)	
Drinking bar	40 (12.0)	22 (55.0)	18 (45.0)	
*Reasons for drinking alcohol*				
Group culture	107 (32.1)	55 (51.4)	52 (48.6)	0.037^*∗*^
Boost confidence	72 (21.6)	37 (51.4)	35 (48.6)	
Whet appetite for meals	62 (18.6)	42 (67.7)	20 (32.3)	
Taking as medicine	50 (15.0)	21 (42.0)	29 (58.0)	
As part of community/household ceremonial activities	25 (7.5)	13 (52.0)	12 (48.0)	
Forget worries	17 (5.1)	13 (76.5)	4 (23.5)	
*Perceived effects of alcohol consumption*				
Poor performance in school	131 (39.3)	71 (54.2)	60 (45.8)	0.709^*∗*^
Engage in misconduct or deviant acts	120 (36.0)	67 (55.8)	53 (44.2)	
Makes you confident/stronger	24 (7.2)	11 (45.8)	13 (54.2)	
Makes you forget your worries	24 (7.2)	13 (54.2)	11 (45.8)	
Results in accidents and deaths	31 (9.3)	16 (51.6)	15 (48.4)	
Makes you prone to disease infection	3 (0.9)	3 (100.0)	0 (0.0)	
Total	333 (100.0)			

^*∗*^Fisher exact test.

## Data Availability

The raw data for the findings of this study are freely available from the corresponding author upon request.
